# The Hidden Persistence of Deep-Sea Pollution

**DOI:** 10.1021/acs.est.6c03631

**Published:** 2026-04-03

**Authors:** Jordi Dachs, Clara Serrano, Núria Trilla-Prieto, Júlia Grande-Martí, Maria Fernández-Altimira, Jessica Patrone, Naiara Berrojalbiz, Laurence Méjanelle, Maria Vila-Costa

**Affiliations:** † Department of Environmental Chemistry, IDAEA-CSIC, 08034 Barcelona, Catalunya, Spain; ‡ LECOB, UMR CNRS-Sorbonne Université 8222, 66650 Banyuls sur mer, France

**Keywords:** pollution, persistence, deep-sea, ocean, organic contaminant, plastic

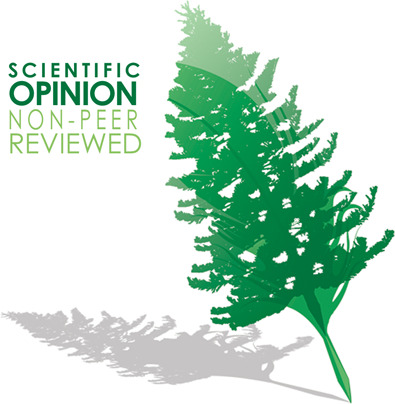

## Graphical abstract

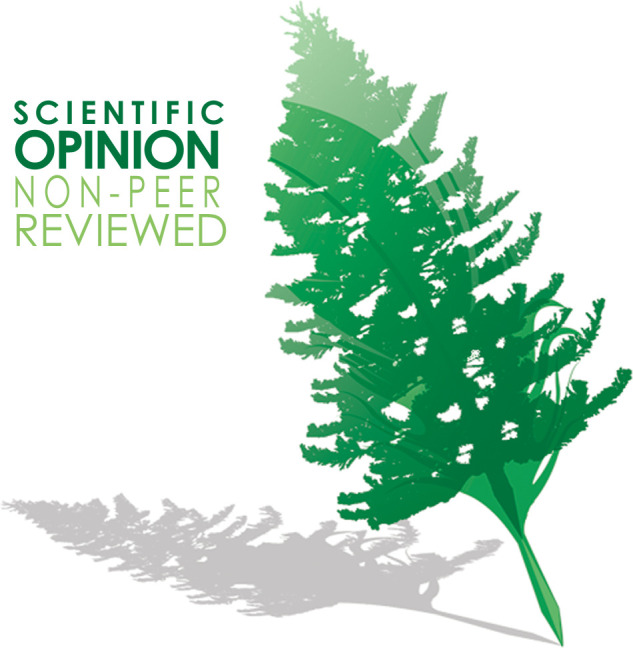

The actual persistence of a chemical is determined not only by its chemical structure but also particularly by its journey once it enters the environment, as it can encounter diverse environmental conditions affecting abiotic and biotic degradation, such as radiation, radical concentrations, or microbial communities. The widespread occurrence of organic contaminants in the ocean, including plastics, is an indicator of their persistence,^1^ as more labile contaminants will be degraded before reaching open sea waters. Over the past several decades, there have been clear advances in our knowledge of the occurrence, transport, and cycling of a wide range of anthropogenic chemicals in the ocean, not only for legacy persistent organic pollutants, such as polychlorinated biphenyls (PCBs), but also for perfluoroalkyl substances (PFAS), organophosphate esters, and plastics, among others.^1–5^ Most of these measurements were taken for the surface ocean, generally in the top several meters of the ocean. These assessments have been used to derive temporal trends of pollution in oceanic regions.^2^ However, this approach neglects the influence of the vertical transport of contaminants,^5–8^ which may result in a decrease in the concentration at the surface with increasing accumulation at depth. Furthermore, the drivers and rates of degradation will vary at different depths and for different biogeochemical provinces.

The vertical journey of contaminants can occur in some marine regions by convection of water masses from the surface to greater depths. During convection, both dissolved and particle phase contaminants will sink, and this process explains the occurrence of deep pollution in some regions, but not generally for the global ocean.^6,7^ Overall, the settling of particle-bound contaminants by the biological pump is thought to be the main process exporting pollutants from the surface ocean.^6^ However, most of the settling organic matter exported from the photic zone is remineralized during its vertical transport, especially in the meso-pelagic zone between 200 and 1000 m depth. A large reduction in the level of particulate/floc organic matter will induce an increase in the fugacity of the contaminant in the particle phase (by solvent depletion) and drive a redissolution of the contaminant (Figure 1). Even though this process has been mentioned previously,^4^ the three-way interactions among contaminants, organic matter transformations, and microbial communities along the water column remain unexplored.

**Figure 1:**
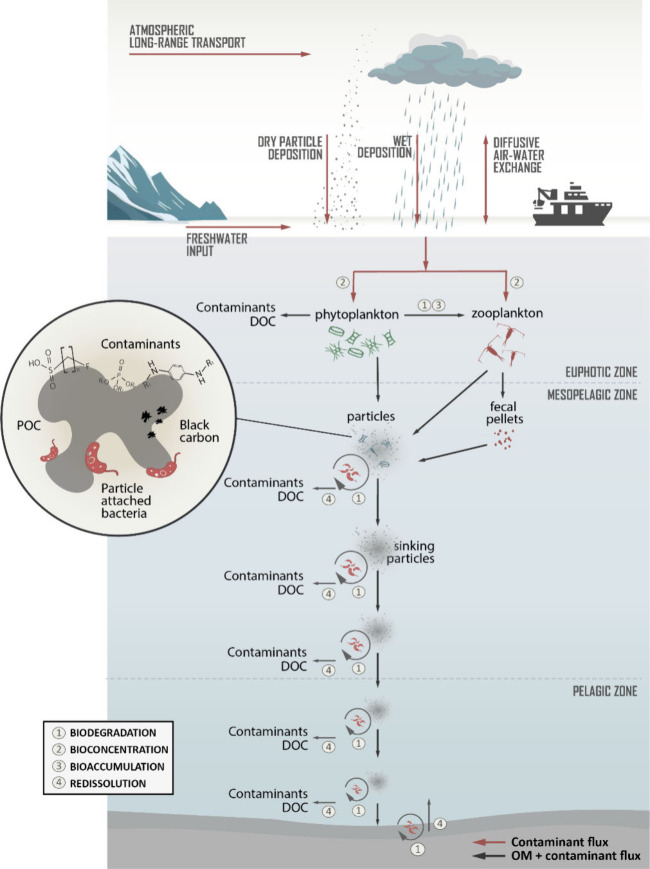
Schematics of the vertical transport of an organic contaminant from the surface ocean to the abyssal depths. The particles and particle aggregates contributing to the biological pump are in part remineralized during the vertical transport. As organic matter degrades, the amplified fugacity gradient between the particle and dissolved phases drives a redissolution of the chemical in deep waters. Only a fraction of the organic contaminant will reach the sediment. After entering deep waters, the chemical will follow a slow cycle of global transport through the global conveyor belt thermohaline circulation.

Despite the sampling difficulties, an increasing number of reports have described the depth distributions of organic contaminants.^3,4,9–11^ In some cases, pollutants follow a surface-enrichment depth-depletion profile, consistent with the inputs of chemicals at the surface by riverine inputs or atmospheric deposition, and their vertical transport by the biological pump due to sorption of contaminants to detritus, plankton, and fecal pellets, as well as by flocs of organic matter (Figure 1). However, the available observations sometimes deviate from this trend, with subsurface maxima, and even with concentrations of some contaminants in pelagic depths that compare or exceed those at the surface.^3,4,11^ In addition, some studies report extremely high concentrations of organic contaminants in biota at depths of several thousand meters,^9,12,13^ implying very high concentrations of pollutants in the abyssal ocean.

The ocean is not only one of the largest reservoirs of pollution but also the main sink for many contaminants. The marine environment holds an enormous diversity of microorganisms that harbor broad metabolic capacities for degradation of organic compounds, which also have the potential to degrade many anthropogenic contaminants through both metabolic and cometabolic processes. Organic contaminants in the oceans exhibit a wide persistence spectrum, ranging from labile compounds that degrade microbially in surface waters, such as some polycyclic aromatic hydrocarbons, to highly recalcitrant nano- and microplastics or PCBs that last decades or centuries. However, microbial activity is reduced at depth, with much lower bacterial and fungal abundances, and also with bacterial production that is several orders of magnitude lower than that at the surface.^14^ Such reduced microbial activity and biodegradation imply that chemical pollution is more persistent at depth than in the photic ocean. Pollution reaching abyssal sediments and waters may remain immobilized and thus become a definitive sink in the long term, even if the chemical is not degraded. However, the fraction of contaminants that redissolves in the meso-pelagic and deep waters of the water column (Figure 1) initiates a slow cycle associated with oceanic circulation that further amplifies the long-term impact of chemical persistence.

The global conveyor belt drives the large-scale and long-term thermohaline circulation in the global ocean.^15^ Under this circulation, deep water formation by subduction occurs at high-latitude regions of the Labrador Sea and Norwegian Sea in the North Atlantic/sub-Artic, or the Weddell Sea and Ross Sea in the Southern Ocean. Deep water masses undergo a global slow movement through the Atlantic Ocean, Southern Ocean, and Indian Ocean to eventually rise to the surface in the Pacific Ocean. Once at shallower depths, they will follow the surface currents. This circulation is key for the transfer of matter and energy at regional and global scales, but its implications remain largely unexplored for organic contaminants. The turnover time of the global conveyor belt, thus the time needed for a chemical from when it undergoes subduction to the deep ocean until it returns to the surface, is 1000–2000 years.^15^ During most of this journey, the water mass and associated contaminants are in the deep ocean, precisely where there is low microbial activity and no photochemistry. For chemicals reaching deep waters in water masses to be upwelled over a longer time, the turnover times and the time spent in deep environments would be longer, ranging from centuries to millennia, as opposed to those of chemicals reaching the deep sea in regions closer to upwelling regions, having turnover times of decades to centuries.^15^ The deep-sea stability of contaminants, such as PCBs, PFAS, or nanoplastics, is unknown at these time scales. Contemporaneous contaminants and those from our environmental past could resurface in the next several decades, centuries, or millennia, thus affecting the planetary health, from marine biota and ecosystems to human health, of future generations. These anthropogenic fingerprints and impacts are cumulative, in the sense that past, present, and future contaminants will contribute to this environmental perturbation of the future composition of the biosphere.

This scenario places chemical persistence at the center of risk assessment when transgenerational impacts are considered and highlights the need to address deep marine pollution while improving our understanding of the factors controlling which chemicals are more likely to reach the deep ocean and persist over long time scales. Future research should include vertically resolved measurements of pollutants and organic matter transformations in both dissolved and particle phases, coupled with the characterization of the particle-associated and free-living microbial community structure and function in the deep sea. Integrating microbiological processes is critical to assess depth-dependent metabolic pathways under such restrictive conditions. Contaminant degradation should be evaluated *in situ* or under conditions mimicking the pressure, temperature, and biogeochemical conditions found in the deep sea. The integration of predictive biogeochemical and circulation modeling approaches will also be essential to assess the persistence and behavior of organic contaminants in the long term and under future environmental scenarios. Such knowledge from field, experimental, and modeling research efforts is required to eventually undertake the appropriate policy measures on individual anthropogenic chemicals or classes of them.
